# Real-world evaluation of an ambient AI scribe in Spanish outpatient care after 2.3 million uses: impact on clinician experience, semantic agreement, and workflow efficiency

**DOI:** 10.3389/fdgth.2026.1874919

**Published:** 2026-07-06

**Authors:** Jose María Alcázar-Peral, Juan Antonio Álvaro-de la Parra, Daniel Blanco, Ángel Blanco, María Elvira Barrios, Ion Cristóbal, Cristina Caramés

**Affiliations:** 1 Corporate Department of Healthcare and Research, Quirónsalud Healthcare Network, Madrid, Spain; 2 General Operations Directorate, Quirónsalud Healthcare Network, Madrid, Spain; 3 Escuela de Doctorado UAM, Centro de Estudios de Posgrado, Universidad Autónoma de Madrid, Madrid, Spain; 4 Information Systems Department, Quirónsalud Healthcare Network, Madrid, Spain; 5 Programa de Doctorado en Medicina y Cirugía, Universidad Autónoma de Madrid, Madrid, Spain.

**Keywords:** ambient AI scribe, burnout, clinical documentation, professional experience, semantic agreement, usage rate

## Abstract

**Introduction:**

Ambient artificial intelligence (AI) documentation systems have emerged as a promising strategy to reduce electronic health record burden and support patient-centered, value-based healthcare (VBHC). Early studies report gains in efficiency and clinician well-being, but large-scale evaluations outside North America are limited. We aimed here to assess the 16-month real-world implementation of an ambient AI documentation system (Scribe) in outpatient care in Spain, focusing on its adoption, transcription semantic agreement, efficiency, and clinician experience.

**Methods:**

A retrospective, multicenter observational analysis was performed using aggregated operational data from September 2024 to December 2025. Monthly Scribe adoption, consultation duration by modality, and cosine-similarity–based semantic agreement were evaluated. Professional experience was assessed through two cross-sectional surveys, including usage-intensity stratification. Statistical analyses included descriptive metrics, Welch tests, Hedges' g, FDR correction, and reliability and correlation analyses.

**Results:**

Scribe adoption increased from 2.7% to ∼31% of all outpatient visits, totalling more than 2.33 million assisted encounters. Consultation duration showed modest differences (15.01 vs. 14.65 min), with several months favouring Scribe and convergence toward parity as workflows matured. Semantic agreement remained high and stable (87.4–89.2%). Professional experience showed improvements in five of seven domains (g = 0.25–0.41) with excellent reliability (α > 0.93) and consistent gains across usage groups.

**Conclusion:**

Large-scale deployment of ambient AI documentation was feasible, showed high semantic agreement, was well-accepted and was associated with improvements in clinician experience. Maturation patterns suggest that efficiency gains may be reinvested into patient interaction and outcome-relevant care, consistent with a VBHC-aligned hypothesis.

## 1 Introduction

Clinical documentation remains one of the most persistent sources of administrative burden in modern healthcare, with physicians routinely spending substantial portions of their working hours on electronic health record (EHR) tasks rather than direct patient care (1–3). This imbalance contributes to reduced clinical efficiency, higher cognitive load, and professional burnout, ultimately affecting quality of care (4, 5). As healthcare systems expand their digital infrastructures, interest has grown in technological solutions capable of reducing clerical load while preserving the accuracy, completeness, and safety of clinical records. In this context, value-based healthcare (VBHC) emphasizes the optimization of health outcomes and patient experience relative to the resources used, focusing on efficiency, quality of care, and patient-centered outcomes.

Ambient artificial intelligence (AI) scribes—systems that capture and structure the clinical encounter using automatic speech recognition, natural language processing, and large language models—have emerged as a promising response to this challenge (6–8). Early evidence from large U.S. networks shows reductions in documentation time, and declines in after-hours work, accompanied by gains in clinician satisfaction and perceived visit quality (9–12). Recent randomized and quasi-experimental studies further indicate that these systems can enhance workflow efficiency and reduce administrative burden without compromising data fidelity (13–15). Despite this growing body of evidence, most implementations to date have been concentrated in North America, and large-scale European deployments remain scarce.

At the same time, concerns persist regarding accuracy, reliability, interoperability, and regulatory compliance, particularly as generative AI becomes increasingly integrated into clinical workflows. Recent assessments highlight the variability in transcription quality across specialties, the need for systematic validation frameworks, and the importance of governance structures that ensure responsible integration, transparency, and clinical oversight (16–18). Furthermore, the impact of ambient AI scribes on patient-informed experience—a key component of VBHC—remains an understudied dimension in real-world European settings. Within this framework, the outcomes evaluated in this study can be interpreted as proxies of VBHC dimensions: consultation duration as an indicator of workflow efficiency, semantic agreement as a proxy for documentation quality and safety, and clinician experience as a contributor to patient-centered care.

In this context, the Scribe Project at Quirónsalud represents one of the first large-scale deployments of an ambient AI documentation system in European outpatient care. Following more than 2.3 million uses across a broad range of specialties, the project offers an opportunity to examine longitudinal patterns of adoption, semantic agreement, consultation duration, and both clinician- and patient-reported experience. The present study provides a detailed evaluation of system performance over time, including temporal trends in semantic agreement, workflow efficiency and professional experience. This work aims to contribute empirical evidence on the real-world impact and maturity trajectory of ambient AI scribes in a European healthcare environment, informing responsible scaling in clinical practice.

## 2 Methods

### 2.1 Study design

We conducted a multicenter retrospective observational study assessing the real-world deployment of an AI-assisted ambient documentation system (Scribe) across outpatient services from September 2024 to December 2025. The study included all hospitals and outpatient medical centers in the Quironsalud network where Scribe was active during the study period. All specialties using the tool in routine outpatient care were incorporated. Scribe was made available for routine use across outpatient consultations without predefined restrictions on visit type or patient characteristics. Its use was not mandated and depended on clinician preference and workflow integration. As the analysis relied on complete aggregated corporate data, no sample size calculation was necessary. No major changes in EHR workflows or organizational processes occurred during the study period that would be expected to significantly influence the evaluated outcomes.

### 2.2 Data sources

The study used aggregated operational data and anonymized survey sources. Outpatient activity datasets provided total consultations, Scribe-assisted visits, and average consultation durations. Semantic agreement was obtained from Scribe's internal cosine-similarity validation workflow comparing AI-generated and clinician-confirmed fields. Clinician experience data came from two structured cross-sectional surveys covering seven domains of perceived impact. All datasets were fully aggregated and contained no identifiable patient information.

### 2.3 Scribe system architecture

Scribe is an in-house developed ambient clinical documentation system based on a modular pipeline integrating automatic speech recognition (ASR) and large language model (LLM) components. Audio from the clinical encounter is transcribed and subsequently processed to extract clinically relevant information and generate structured reports based on predefined templates. The system supports multilingual clinical environments with automatic language identification and is integrated with the electronic health record (EHR), allowing clinicians to review and edit generated content prior to validation. During the 16-month observation period, the system underwent iterative updates to its underlying ASR and LLM components as part of routine deployment. These updates, which did not modify the overall architecture, should be considered when interpreting longitudinal performance trends.

### 2.4 Usage rate and consultation duration analyses

Monthly adoption of the Scribe consultation model was assessed using aggregated corporate activity data from September 2024 to December 2025. For each month, the dataset included the total number of outpatient consultations and the number of consultations assisted by Scribe, from which the monthly usage rate (%) was calculated as follows: Usage Rate = (Scribe Consultations/Total Consultations) × 100. We also examined month-over-month (MoM) changes in Scribe activity, both in absolute numbers and percentage growth, to characterize the adoption rate. Cumulative Scribe consultations were obtained by sequentially summing the monthly values, allowing us to visualize long-term adoption patterns over the study period. To evaluate consultation duration over the full September 2024–December 2025 period, we computed period weighted means for each modality, using the number of monthly consultations as weights. Monthly differences between modalities were calculated as the difference between average Scribe duration and average non-Scribe duration. This measure enabled month-by-month identification of whether Scribe-assisted consultations were faster, slower, or equivalent to standard consultations.

### 2.5 Semantic agreement and evaluation procedure

The semantic agreement of Scribe's generated documentation was assessed by comparing the cosine similarity between each AI-generated field and the corresponding field validated by the physician within the structured consultation form. In this workflow, the clinician-validated field represents the final version after review and, when necessary, editing of the AI-generated draft. This metric therefore reflects the degree of semantic agreement between the AI-generated output and the clinician-validated documentation, rather than transcription accuracy against an independent ground truth. After the physician reviews and, if necessary, edits the automatically generated text, both the AI output and the physician's final text are converted into vector embeds within Scribe's internal NLP model. Cosine similarity is calculated using the standard formulation: Similarity(A,B)= (A⋅B)/∥A∥∥B∥, where A⋅B represents the dot product, while ∥A∥ and ∥B∥ denote vector quantities. Cosine similarity was calculated using the final clinician-validated version of each field after completion of the review and editing process.

All indicators contribute equally to the query-level score. For categorical or list-based fields, similarity is set to 0% when the clinician's selected option differs from the AI-generated one. Therefore, the overall query semantic agreement is calculated as the unweighted mean of the N field-level similarities. Cosine similarity has been widely used in clinical NLP tasks to assess the correspondence between AI-generated and clinician-created narratives, including applications in automated ICD-10 coding and radiology report comparison (19–21).

### 2.6 Professional experience evaluation

Two cross-sectional clinician surveys were administered in May 2025 (Round 1) and July 2025 (Round 2). The full survey instrument is provided as Supplementary Material (original Spanish version with English translation). Round 1 included all clinicians with more than 20 Scribe-assisted consultations, whereas Round 2 included clinicians with more than 50 consultations and further stratified respondents by cumulative Scribe use: advanced (>300 consultations), medium (150–300), and basic (>50). In Round 1, 1,255 invitations were distributed and 184 responses were collected (response rate: 14.7%). In Round 2, 2,093 invitations were distributed across predefined usage groups, yielding 163 responses overall (response rate: 7.8%). Response rates by subgroup were 9% (advanced users), 9.6% (medium users), and 5.7% (basic users). Emergency departments were excluded. Participants rated seven domains related to professional experience on a 0–10 Likert scale (higher scores indicating more favorable perceptions). A composite experience score was computed as the respondent-level mean across the seven domains. Additional demographic and open-ended questions were included in the survey but are not analyzed in the present study. The two survey rounds were conducted as independent cross-sectional assessments and were not designed as paired longitudinal measurements of the same individuals.

### 2.7 Statistical analysis

Descriptive statistics included mean, SD, SE, and 95% CI. When analyzing professional experience, differences between rounds were assessed using Welch's *t*-test, and standardized effect sizes were quantified using Hedges' g (22). Multiple comparisons were controlled using the Benjamini-Hochberg FDR test (5%). The internal consistency of the seven-item instrument was assessed using Cronbach's α, following established psychometric guidelines (23). Structural stability between rounds was assessed using Pearson's r and Spearman's ρ of the domain-level means (24). Differences between groups in intensity of use of the second round were analyzed using omnibus ANOVA or Kruskal–Wallis tests.

### 2.8 Ethics and reporting standards

The study adhered to the principles of the Declaration of Helsinki and received formal approval from the Research Ethics Committee of the Jiménez Díaz Foundation University Hospital (ref. EO299-24). All analyses were conducted using fully anonymized, aggregated datasets, and no identifiable personal information or clinical interventions involving patients were required. Patients were informed about the use of the Scribe system during the clinical encounter, and verbal consent was obtained for its activation. When consent was granted, the consultation is done using Scribe, and the patient's verbal consent is documented within the consultation transcript. The processing of recorded clinical conversations was conducted in accordance with applicable data protection regulations, including GDPR Article 9, with verbal informed consent obtained from patients and documented. In cases where patients declined its use, the consultation proceeded without Scribe, following standard clinical practice. Clinicians remained responsible for reviewing and validating all generated clinical documentation before its inclusion in the electronic health record.

## 3 Results

### 3.1 Scribe usage rate

**Figure 1:**
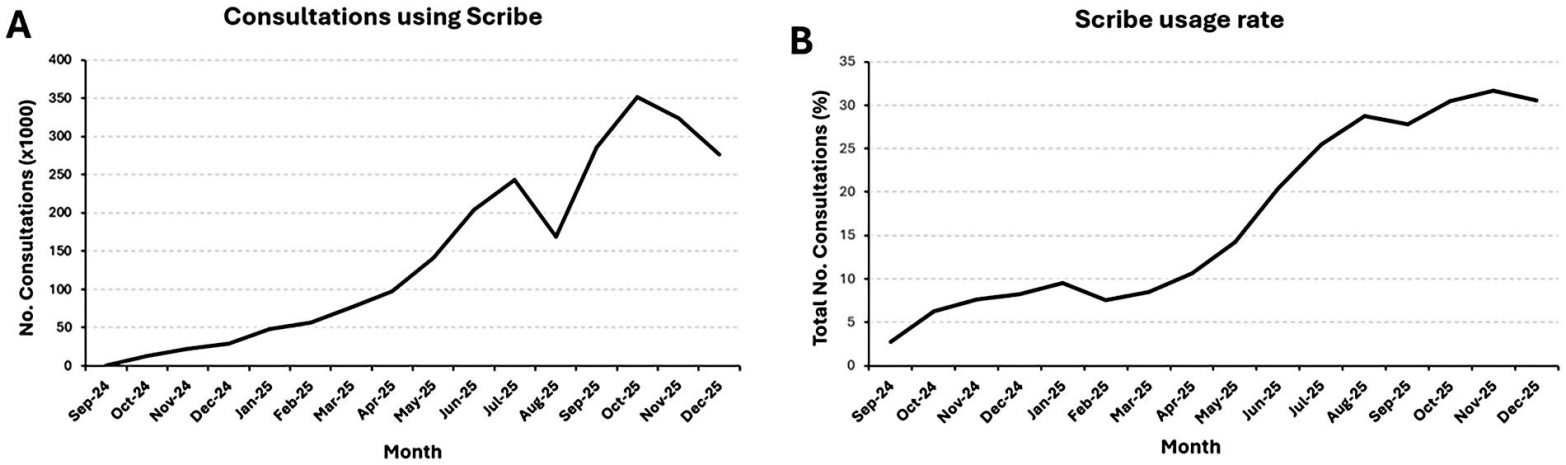
Cumulative monthly scribe consultations **(A)** and scribe usage rate **(B)** from September 2024 to December 2025.

Between September 2024 and December 2025, a total of 11,599,484 outpatient visits were recorded, of which 2,339,281 were completed using Scribe, representing an overall usage rate of 20.17%. Monthly adoption increased steadily from an initial 2.72% in September 2024 to sustained levels above 30% by the end of 2025, peaking at 31.62% in November and remaining high in December (30.55%) (Figure 1). This growth trajectory reflects a clear expansion from initial implementation to widespread integration across all services, with average monthly usage reaching 16.91% (SD: 10.54). Cumulative activity exceeded 2 million consultations assisted by Scribe by the fourth quarter of 2025, illustrating strong and continued adoption as the tool became integrated into routine medical practice.

### 3.2 Evolution of the number of users

**Figure 2:**
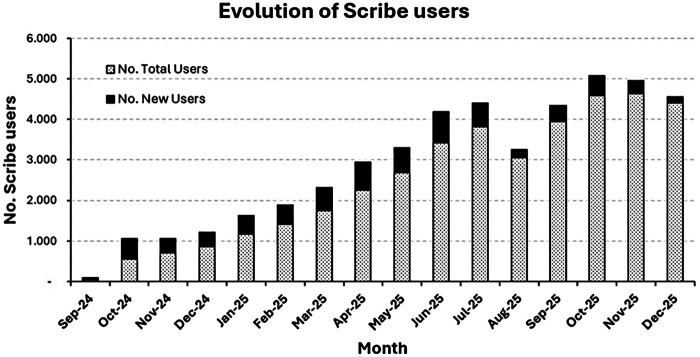
Temporal evolution in the number of total and new scribe users.

The number of clinicians actively using Scribe increased steadily, from 45 users in September 2024 to a peak of 4,633 users in October 2025, followed by a slight stabilization at 4,397 users in December 2025 (Figure 2). Monthly onboarding was particularly strong during the expansion phase in early to mid-2025, with several months exceeding 500 new users, including 690 new users in April and 758 in June. Two temporary seasonal dips were observed: August 2025 (205 new users) and, to a lesser extent, December 2025, both coinciding with typical holiday periods. Overall, these trends reflect rapid and sustained growth in the active user base during the implementation period.

During the study period, a total of 7,147 clinicians used Scribe at least once, of whom 3,506 performed more than 100 queries and 1,617 exceeded 500, indicating deep and substantial adoption over time. Users exceeding 100 monthly uses grew steadily, reaching 947 in July 2025, while those exceeding 500 monthly uses also increased steadily, peaking at 34 in July 2025, before showing expected declines during holiday periods (Supplementary Figure S1). These results demonstrate not only broad adoption but also a sustained increase in regular, high-intensity use, reflecting increasingly deep integration of Scribe into clinical workflows.

### 3.3 Changes in efficiency

**Figure 3:**
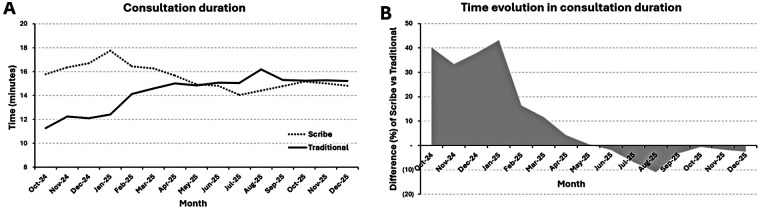
Analysis of consultation duration. Monthly mean consultation duration by modality **(A)**, and evolution of difference in consultation duration (Scribe minus non-Scribe) **(B)**.

Throughout the observation period, the weighted mean consultation duration was 14.72 min, with Scribe-assisted consultations averaging 15.01 min and non-Scribe consultations averaging 14.65 min, a difference of +0.37 min. Monthly values showed marked variability: at the beginning of the timeline, Scribe-assisted consultations were consistently longer than non-Scribe consultations, while from mid-2025 onward, several months showed shorter durations with Scribe (e.g., June–August 2025). In the final months of 2025, the difference between the modalities decreased, and similar durations were observed (e.g., October and December 2025) (Figure 3). During the 16-month period, seven months showed faster Scribe-assisted consultations, while in the remaining months, Scribe durations were similar or slightly longer.

### 3.4 Analysis of semantic agreement

**Figure 4:**
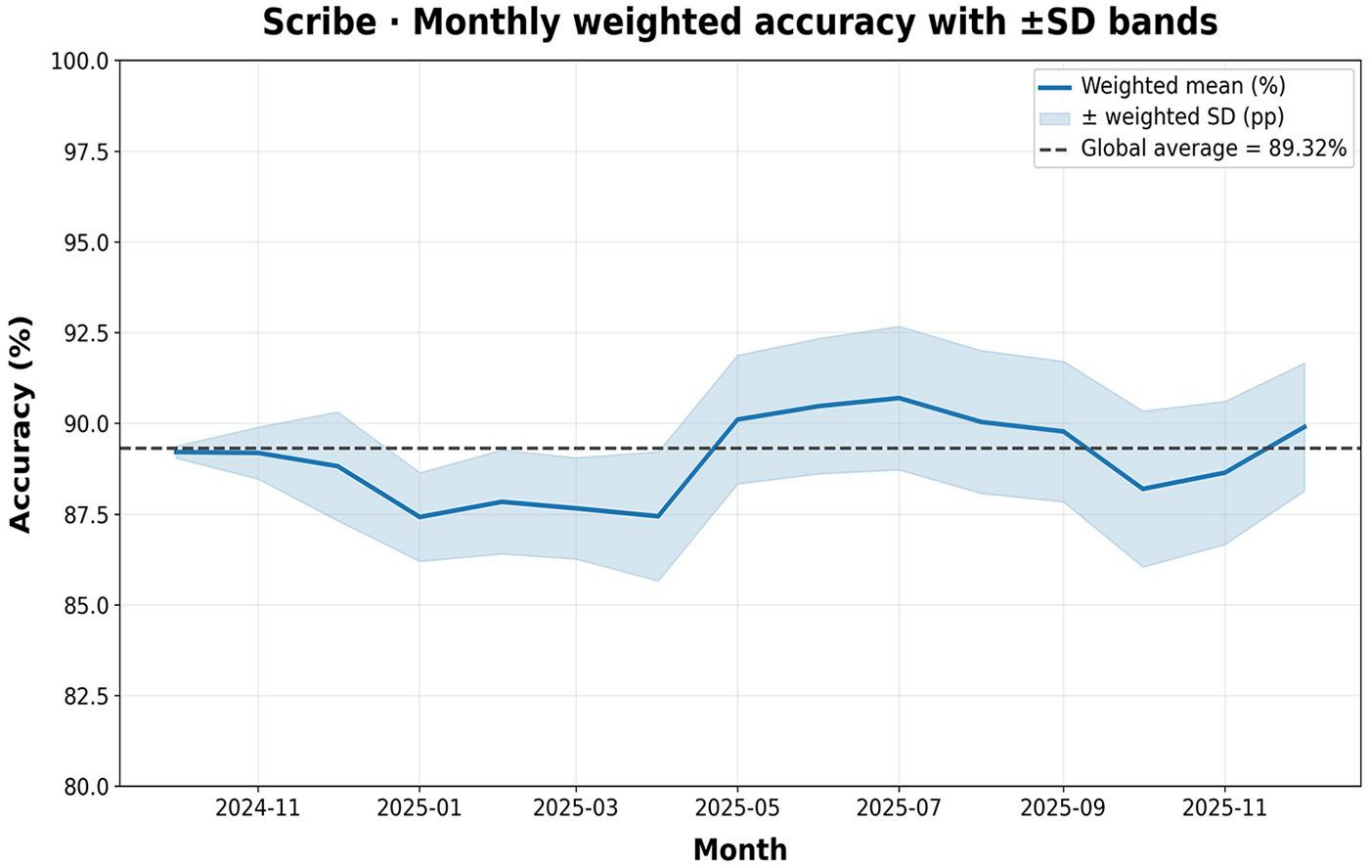
Monthly weighted accuracy over time. The central blue curve represents the weighted mean accuracy per month, while the shaded band indicates ± weighted standard deviation, capturing within-month variability. A dashed horizontal line marks the global weighted average accuracy.

Time-series analysis showed remarkable temporal stability in Scribe's semantic agreement throughout the observation period (Figure 4). Weighted monthly values ranged within a narrow range of 87.4% to 89.2%, with standard deviation bands typically within ±1–1.5 percentage points, indicating minimal month-to-month fluctuation. When plotted on a fixed scale of 80% to 100% on the *Y*-axis, the trend appeared visually flat, reinforcing the consistency of the system's performance despite fluctuations in seasonal workload, case mix, or the gradual onboarding of new specialties.

A horizontal reference line representing the overall weighted average (89.32%) showed that almost all months clustered closely around this benchmark, with no evidence of systematic deviation. Small deviations coincided with periods of higher service adoption, during which greater heterogeneity in documentation styles temporarily amplified the variance. Overall, longitudinal analysis indicates that Scribe maintained high and stable semantic agreement throughout the implementation period.

Specialty-stratified analyses (Supplementary Table S1) showed that adoption rates, semantic agreement, and consultation duration patterns were broadly consistent across specialties, although some variability was observed. Differences in duration ranged from slight increases in some specialties to modest reductions in others. Semantic agreement ranged from 84.7% (Internal Medicine) to 92.6% (Otolaryngology), while consultation duration differences varied from approximately −1.9 to +3.7 min, reflecting moderate heterogeneity depending on clinical context.

### 3.5 Evaluation of professionals' experience using scribe

Next, we assessed professionals' experience with Scribe through two rounds of anonymous surveys covering seven domains of perceived impact. The first survey (Round 1), conducted in May, included all clinicians with at least 20 consultations assisted by Scribe. The second survey (Round 2), conducted in July, incorporated the same seven-domain instrument and stratified participants into three usage intensity groups (advanced, intermediate, and basic) based on the cumulative number of consultations using Scribe. This structure allowed us to evaluate both the evolution of professional experience over time and any differences associated with the level of exposure to the tool.

Across the seven domains assessed, standardized effect sizes indicated small to moderate improvements between Round 1 and Round 2 (Table 1 and Supplementary Figure S2). The greatest improvements were observed in learning through the algorithm (g = 0.41) and transcription quality (g = 0.37), followed by significant improvements in administrative burden and stress/quality of life (g = 0.30). Job satisfaction also increased (g = 0.25). Domains with high baseline scores (doctor-patient interaction and patient care) showed minimal change (g = 0.14 and g = 0.13). The composite experience score showed consistent overall improvement (g = 0.32), indicating a positive overall change between rounds.

**Table 1:** Professional experience by domain: round 1 (*n* = 184) vs. Round 2 (*n* = 163).

	Round1		Round 2				
Domain	Mean (SD)	CI^a^95%	Mean (SD)	CI95%	Welch *p*-value	Hedges' *g* (CI95%)	*q-*value (FDR^b^)
Administrative workload	4.26 (2.97)	3.83–4.69	5.16 (3.06)	4.69–5.63	0.006	0.30 (0.09–0.51)	0.014
Doctor–patient interaction	6.45 (2.65)	6.07–6.83	6.82 (2.53)	6.43–7.21	0.184	0.14 (−0.07–0.35)	0.184
Learning by the algorithm	4.84 (2.77)	4.44–5.24	5.98 (2.75)	5.55–6.40	<0.001	0.41 (0.20–0.62)	<0.001
Transcription quality	4.13 (2.61)	3.75–4.51	5.06 (2.41)	4.69–5.43	0.001	0.37 (0.16–0.58)	0.004
Stress/quality of life	4.20 (2.99)	3.76–4.63	5.11 (3.07)	4.64–5.58	0.005	0.30 (0.09–0.51)	0.012
Patient care	5.55 (2.80)	5.15–5.96	5.93 (2.93)	5.48–6.38	0.229	0.13 (−0.08–0.34)	0.229
Professional satisfaction	4.78 (3.05)	4.34–5.22	5.53 (3.04)	5.07–6.00	0.021	0.25 (0.04–0.46)	0.030
**Composite**	**4.89** (**2.42)**	**4.54–5.24**	**5.66** (**2.41)**	**5.29–6.03**	**0**.**003**	**0.32** (**0.11–0.53)**	**0**.**010**

^a^CI, confidence interval; ^b^FDR, false discovery rate.

Notably, the professional experience instrument demonstrated excellent internal reliability in both rounds (Cronbach's *α*= 0.937 in round 1; *α*= 0.935 in round 2), confirming strong internal consistency and stability across all measurement rounds. Moreover, cross-round correlation analyses showed that the profile of domain scores remained largely unchanged (Pearson's r = 0.962; Spearman's *ρ*= 0.964), indicating a stable underlying structure. All seven domains increased from Round 1 to Round 2, supporting pattern replication and suggesting a consistent maturation effect, rather than structural changes. A sign test confirmed complete directional agreement (7/7 domains increasing).

Inter-round testing revealed statistically significant improvements in five of the seven domains and in the composite score after FDR correction. The domains showing the most significant and statistically robust increases were Algorithm Learning, Transcription Quality, Administrative Burden, and Stress/Quality of Life. Doctor-Patient Interaction and Patient Care, which already had high baseline scores, showed no significant differences after correction.

In Round 2, comparisons among advanced, medium, and basic users revealed only limited subgroup differences. Although an omnibus ANOVA detected a difference in Learning by the algorithm (*p* = 0.017), no subgroup differences emerged for the remaining domains or for the composite score (composite omnibus *p* = 0.534). These findings suggest that, while higher exposure may modestly influence perceived algorithm learning, overall improvements in professional experience were broadly consistent across usage groups (Supplementary Table S2 and Supplementary Figure S2).

## 4 Discussion

Over a 16-month period (September 2024–December 2025), ambient AI documentation (Scribe) scaled from 2.7% to ∼31% of all outpatient consultations, surpassing 2.33 million Scribe-assisted encounters. This trajectory extends our initial implementation window, indicates broad, sustained adoption across services and this pattern is notable when compared with the typical diffusion timelines of digital health tools integrated into electronic health records (EHRs), which often require several years to achieve similar penetration, particularly when use is not mandatory. Achieving this transition is particularly challenging in healthcare settings, where professional autonomy, risk aversion, and heterogeneity of clinical practice tend to slow the spread of innovations (25). In concordance with prior health-system evaluations, clinicians typically report lower documentation burden, more same-day closure, and reduced time-in-note, albeit with heterogeneity by specialty, baseline documentation load, and utilization intensity (5, 9, 16, 26–30). Such variability underscores a VBHC-aligned perspective: technology should return time to high-value clinical interactions and patient-relevant outcomes, rather than uniformly shortening consultations times.

During the study period, no major organizational, workflow, or documentation policy changes were identified that would be expected to substantially confound adoption or efficiency outcomes. Minor adaptations to EHR documentation templates were implemented prior to deployment to facilitate structured reporting, but these did not affect clinical workflows, consultation processes, or documentation requirements. Consultation duration in our series showed a modest overall difference (weighted mean 15.01 min for Scribe vs. 14.65 min for non-Scribe; *Δ* = + 0.37 min), with seven months exhibiting shorter Scribe consultations and convergence toward parity in late 2025. The variability observed across specialties in consultation duration further supports the descriptive nature of these findings (Supplementary Table S1). Differences ranged from slight increases in some specialties to modest reductions in others, likely reflecting heterogeneity in case-mix, documentation practices, and workflow integration. These results suggest that the impact of ambient documentation tools on efficiency may depend on the specific clinical context and stage of adoption, rather than representing a uniform effect across all settings. This pattern aligns with broader evidence showing that administrative and EHR-related time often decreases with ambient documentation tools even when appointment length and throughput remain unchanged during early implementation—a transitional phase typically shaped by review and edit demands, note-length expansion, and learning-curve dynamics (5, 9, 27–29, 31–33). Randomized and quasi-experimental studies further indicate that benefits accumulate gradually over weeks to months and vary by specialty, with improvements in same-day closure and reductions in after-hours work emerging over time (33, 34). From a value-based healthcare perspective, such evolution is desirable: efficiency gains can be reinvested into listening, shared decision-making, and deeper patient engagement, rather than merely compressing consultation time. As Scribe use was not standardized across all encounters and depended on clinician adoption, some degree of selective use at the individual level cannot be excluded. This may have influenced efficiency outcomes and could partly explain the relatively modest differences observed in consultation duration.

Furthermore, as noted above, the metric used in this study captures semantic agreement between AI-generated and clinician-validated documentation within a human-in-the-loop workflow, rather than transcription accuracy against an independent ground truth. While this approach captures real-world system performance, it may overestimate agreement compared to fully external validation frameworks. Our findings show that Scribe maintains consistently high agreement with physician-validated documentation, with global weighted values near 89% and minimal variability across both specialties and time. This level of stability aligns with prior work demonstrating that cosine-similarity–based text comparison is a robust method for evaluating the semantic fidelity of automatically generated clinical documentation. Cosine similarity has been widely applied to clinical narratives—such as the automated ICD-10 encoding of electronic health records in Silva et al. (19) and the semantic alignment of generated radiology reports in Gholipour Picha et al. (21)—with similarly strong concordance between AI-generated and clinician-authored text when structured clinical content is present. Taken together, these results position Scribe's semantic agreement within the upper range reported for AI-assisted clinical documentation systems and support the use of cosine similarity as an appropriate, sensitive metric for monitoring semantic agreement in routine outpatient care.

The professional experience showed excellent reliability across waves (α > 0.93), consistent with established standards for multi item scales in healthcare research (23). Domain profiles were highly stable between rounds, with near perfect correlations, supporting longitudinal measurement stability (24). Despite this structural consistency, five domains improved significantly with small to moderate effects (g = 0.25–0.41), particularly in algorithm learning, transcription quality, administrative workload, and stress/quality of life—patterns aligned with maturation effects described in digital scribe literature (35). Importantly, these gains were broadly consistent across usage intensity groups, indicating that the benefits of Scribe were widely shared regardless of prior exposure, with only modest advantages for higher frequency users. An additional nuance emerges from the analysis by usage intensity (Supplementary Table S2), where a non-monotonic pattern is observed, with intermediate users showing slightly lower scores compared to both basic and advanced users. Several explanations may account for this finding. First, selection effects may play a role, as users progressing to higher levels of adoption could represent a subgroup with greater affinity or perceived benefit. Second, differences in specialty composition across usage strata may contribute to variability in perceived experience. Finally, this pattern may reflect a “trough of disillusionment” among intermediate users, whereby initial expectations are recalibrated before longer-term benefits are fully realized. These interpretations remain speculative but highlight the importance of considering heterogeneity in adoption trajectories.

Taken together, our findings suggest that ambient clinical documentation tools such as Scribe have the potential to achieve meaningful scale in routine medical practice within a relatively short period, if they are well integrated into the EHR and aligned with clinician priorities. Also, our findings are consistent with a maturation model: initial inefficiencies (review/edit diligence, longer notes, confidence building) give way to operational stabilization, with semantic agreement maintained, documentation time reduced, and professional experience signals improving. We therefore view ambient documentation less as a tool to shorten visits and more as an enabler of VBHC—reallocating time toward relationship-centered care, communication, clinical reasoning safety checks, and shared decisions, rather than documentation tasks. From a VBHC perspective, these findings can be interpreted across three dimensions: efficiency (consultation time and workflow integration), quality and safety (semantic agreement), and patient-centered care (clinician experience). Thus, Scribe may function as a potentially value-enabling intervention that preserves face-to-face aligning with contemporary frameworks that define value as outcomes and experience achieved per unit of resource use, rather than speed alone.

Limitations include the observational design and reliance on aggregated operational data for some endpoints, which precludes causal inference and fine-grained analyses of case-mix or clinician-level heterogeneity. The analysis of consultation duration was based on aggregated data and did not allow adjustment at the clinician or visit level. Therefore, the observed differences should be interpreted as descriptive and may be influenced by unmeasured factors such as clinician-specific practices, specialty, or visit type. Future studies using encounter-level data and within-clinician designs would allow a more robust assessment of the impact of ambient documentation tools on efficiency. Nonetheless, the scale and duration of deployment across specialties provide a robust real-world view of ambient documentation's trajectory in a European context. Future work should integrate patient-relevant outcome measures, clinician after-hours/EHR log data, and safety/quality audits to map how documentation efficiency converts into durable VBHC gains. In particular, the VBHC interpretation should be considered hypothesis-generating, as direct measures of patient-reported outcomes, clinical outcomes, and after-hours/EHR use were not available in the present study. In addition, the accuracy metric used in this study reflects semantic agreement between AI-generated and clinician-validated documentation within a human-in-the-loop workflow, rather than transcription accuracy against an independent ground truth. Cosine similarity does not differentiate between types of edits (e.g., transcription errors, clinical corrections, or stylistic changes) and may not fully reflect clinical correctness, as it does not capture omissions, intentional refinements, or the clinical relevance of differences. All fields are weighted equally, which may not reflect the varying clinical importance of different types of information. More broadly, this metric captures semantic agreement rather than clinician editing burden or documentation usability. In addition, the use of clinician-edited text as the reference standard, together with embeddings derived from the same NLP pipeline, may lead to an overestimation of agreement. Future studies incorporating adjudicated clinical audits using structured error severity frameworks would provide a more clinically grounded validation of system performance.

Furthermore, the analysis of clinician experience was based on repeated cross-sectional surveys rather than paired longitudinal data, and therefore observed differences between rounds may partly reflect differences in respondent composition rather than within-individual changes. As no pre-deployment baseline was available, these findings should be interpreted as reflecting changes over time following implementation, rather than causal effects directly attributable to the tool. Another important issue to consider is that response rates were modest, particularly in the second survey round. This may partly reflect the timing of data collection, which took place during the summer period, potentially affecting clinician availability and participation. However, as participation was voluntary, the results may also be subject to self-selection bias, with a possible overrepresentation of more engaged users or those with more favorable perceptions of the tool.

In conclusion, Scribe scaled rapidly while maintaining stable semantic agreement and being associated with improvements in clinician experience. Consultation duration converged toward parity as workflows matured, and efficiency gains were associated with better professional well-being. Framed within value-based healthcare, these observations are consistent with a VBHC-aligned hypothesis, suggesting that ambient AI documentation can return time and attention to patient care, supporting both operational efficiency and patient-centered outcomes.

## Data Availability

The original contributions presented in the study are included in the article/Supplementary Material, further inquiries can be directed to the corresponding authors.
