# Supplementary material for: Real-world evaluation of an ambient AI scribe in Spanish outpatient care after 2.3 million uses: impact on clinician experience, semantic agreement, and workflow efficiency
Source: Front Digit Health. 2026 Jul 6;8:1874919. doi: 10.3389/fdgth.2026.1874919 (PMC13381512; doi:10.3389/fdgth.2026.1874919)
Supplement: Supplementary file 1 [file Datasheet1.pdf]

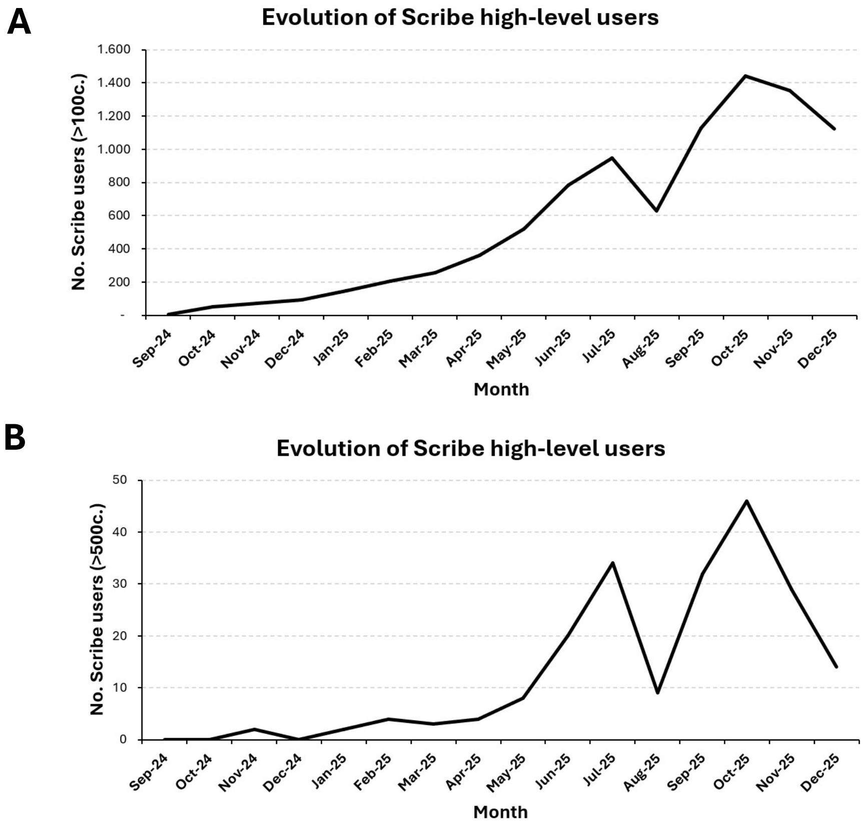

Figure S1. Monthly trend of high-level users with more than 100 (A) and 500 (B) Scribe consultations.

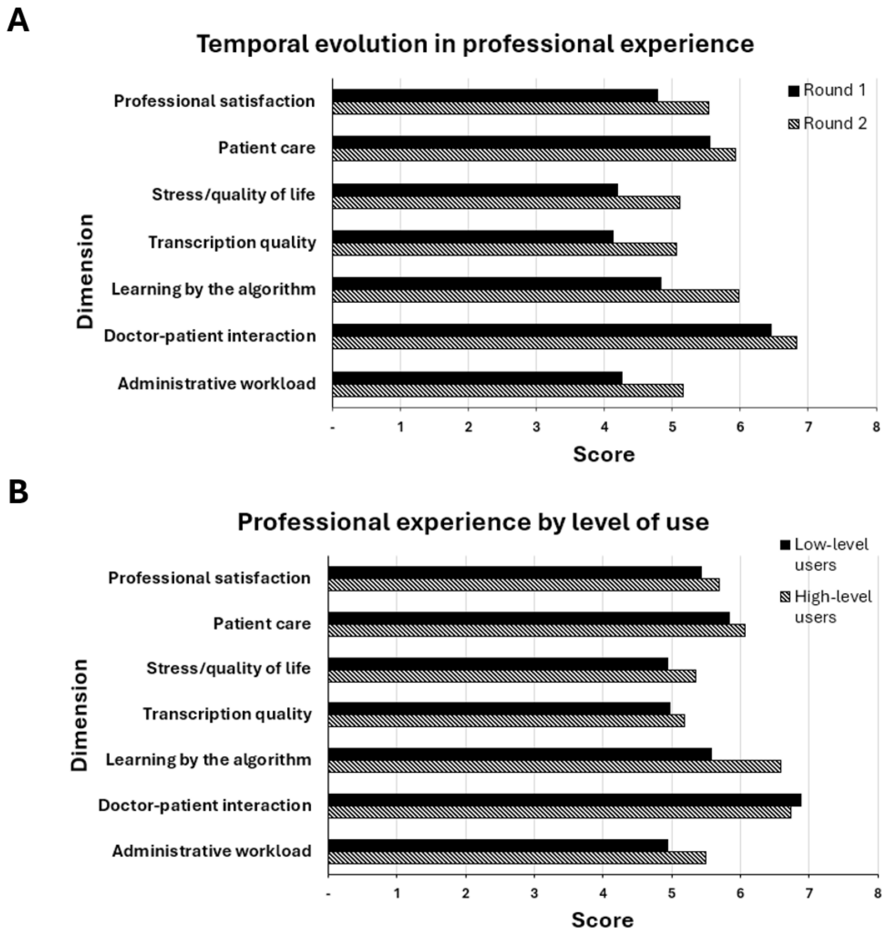

Figure S2. Analysis of the temporal evolution in the professional´s experience using Scribe (A), and by level of use (B).

Table S1. Specialty-stratified performance metrics.

Specialty	Total visits	Usage (%)	Accuracy (%)	Δ Duration (min) (Scribe vs. Traditional)	Months observed
Cardiology	609,078	21.79	89.68	0.52	14
Endocrinology	400,186	16.12	89.49	1.48	14
Gastroenterology	655,247	23.94	89.25	3.68	15
General Surgery	405,613	28.36	91.77	2.14	15
Gynecology	981,318	18.88	87.50	0.22	12
Internal Medicine	384,870	22.80	84.69	-1,91	13
Neurology	348,598	17.06	85.67	-1,46	12
Otolaryngology	676,809	19.76	92.55	2.13	13
Traumatology	2,489,471	23.02	90.08	2.57	16
Urology	565,244	20.74	91.62	1.28	13

Table S2. Round 2 composite score by usage intensity (0–10).

Usage group (Round 2)	n	Mean (SD)	IC95%
Advanced (>300)	65	5.87 (2.71)	5.21-6.53
Medium (150-300)	50	5.36 (2.11)	4.78-5.95
Basic (>50)	48	5.67 (2.28)	5.02-6.31

Supplementary Material 1. Clinician Experience Survey Instrument (Spanish and English versions).

SCRIBE – Formulario de satisfacción (Spanish version)

Datos Demográficos

- ¿Dónde se encuentra el Hospital o Centro Quirónsalud el que desarrollas tu actividad profesional?
- ¿Cuál es tu Servicio/Especialidad?

Por favor, responde a las siguientes afirmaciones en relación a Scribe con una puntación de 0 a 10, siendo 0 “totalmente en desacuerdo”, y 10 “totalmente de acuerdo”:

- La herramienta de pase de consulta asistido por IA (Scribe) permite reducir la carga de trabajo administrativo del profesional médico
- La utilización de Scribe puede mejorar la relación Médico-Paciente
- Percibes una mejora progresiva en el aprendizaje del algoritmo y mejora continua del funcionamiento que se traduce en una menor cantidad de correcciones por tu parte
- Scribe transcribe correctamente la información y genera informes de buena calidad
- El uso de Scribe te puede permitir reducir el estrés, mejorar tu entorno y calidad de la vida laboral
- Esta herramienta ayuda a mejorar la atención ofrecida al paciente
- La implementación de herramientas como Scribe mejora tu satisfacción profesional
- Desde tu experiencia, ¿qué mejoras introducirías en Scribe? (*Opcional. Texto libre*)
- En tu especialidad, ¿en qué procesos es más útil y consideras que tiene mayor aplicación? (*Opcional. Texto libre*)

SCRIBE – Satisfaction Questionnaire

Demographic Data

- Where is the Quirónsalud hospital or center where you carry out your professional activity?
- What is your department/specialty?

Please rate the following statements regarding Scribe on a scale from 0 to 10, where 0 indicates “strongly disagree” and 10 indicates “strongly agree”:

- The AI-assisted consultation tool (Scribe) reduces the administrative workload of the physician
- The use of Scribe improves the doctor–patient relationship
- There is a progressive improvement in the algorithm’s performance over time, resulting in fewer corrections required by the user
- Scribe accurately transcribes clinical information and generates high-quality reports
- The use of Scribe reduces stress and improves the work environment and overall quality of professional life
- This tool contributes to improving the quality of patient care
- The implementation of tools such as Scribe improves professional satisfaction

Optional open-ended questions (not analyzed in the present study)

- From your experience, what improvements would you suggest for Scribe?
- In your specialty, in which clinical processes is Scribe most useful and where do you consider it has the greatest applicability?
